# Computer Assisted Learning: Assessment of the Veterinary Virtual Anatomy Education Software IVALA™

**DOI:** 10.3390/vetsci5020058

**Published:** 2018-06-19

**Authors:** William Brady Little, Elpida Artemiou, Anne Conan, Cathryn Sparks

**Affiliations:** 1 Department of Biomedical Sciences, Ross University School of Veterinary Medicine, P.O. Box 334, Basseterre, Saint Kitts and Nevis; eartemiou@rossvet.edu.kn (E.A.); AConan@rossvet.edu.kn (A.C.); 2 College of Veterinary Medicine, Kansas State University, 228 Coles Hall 1620 Denison Ave, Manhattan, KS 66506, USA; csparks@vet.k-state.edu

**Keywords:** veterinary anatomy education, computer assisted learning (CAL), blended learning, gross anatomy, virtual anatomy, educational methods

## Abstract

Although cadaveric dissection has historically been the cornerstone of anatomical education, it comes at the cost of some emotional, moral, safety, and environmental concerns. Computer assisted learning (CAL) programs are an increasingly common solution to these issues; however, research regarding the efficacy of high fidelity simulation is limited. The traditional first semester veterinary gross anatomy course curriculum at Ross University School of Veterinary Medicine (RUSVM) was supplemented with a web based virtual anatomy program, IVALA™ (www.ivalalearn.com). The purpose of this study was to assess the relationship between supplementary use of the IVALA™ program and student examination scores, and to measure student perception surrounding IVALA™. IVALA™ uses an interactive virtual canine specimen that enables students to identify, move, rotate, magnify, and remove individual anatomic structures while providing a text description of each selected anatomic point. Fifty-six first semester RUSVM students who supplemented their anatomic learning with the IVALA™ program performed significantly higher on examinations compared to students (n = 123) that did not (*p* = 0.003). Students’ overall perception toward IVALA™ was enjoyable (mean = 3.8 out of a 5-point Likert scale) and beneficial to their knowledge of anatomy (mean = 3.7); however, students did not support replacing cadaveric dissection with CAL (mean = 2.1). CAL can effectively supplement learning outcomes for veterinary anatomy.

## 1. Introduction

The understanding of anatomy can be considered as one of the most critical components of medical education, and longest in history of formal medical training [1]. It has been stated that anatomical education is important to all those who interact with patients, and is crucial to fully appreciate embryologic development, surgery, and physiology [2]. Anatomic training is necessary for understanding the three-dimensional body, development of touch mediated perception, appreciating the importance of the patient, learning the basic language of medicine, diagnostic imaging, and medical specialties [3,4]. Veterinary students also appreciate the educational significance of cadaveric dissection as the most useful teaching modality in learning anatomy (54.7%), followed by three-dimensional computer simulation (35.9%), textbooks (4.7%), and lectures (4.7%) [5]. 

Traditional anatomy education techniques including lectures, and anatomical dissection have recently encountered significant challenges and criticism [6,7]. Unequivocally, the laboratory dissection experience is highly demanding of curricular resources as purchasing, utilizing, and disposing of cadavers requires a major time commitment as well as poses a significant financial burden [8,9]. Compounding this problem is the fact that many anatomy course resources are being reduced by budget cuts [10] and less time is being allocated to anatomy teaching [7,11]. Furthermore, external influences such as media and public relations have also increased apprehension about cadaveric usage [12], resulting in a priority of reduction, replacement, and refinement of animal use in education [13]. Other documented concerns in the field of anatomy education include overemphasis of minutia, high student to faculty ratio during laboratory dissections, loss of the clinical relevance, inadequate guidance for students and dependence on rote memorization [6,13,14]. Meanwhile, in numerous institutions, time dedicated to anatomy dissection has been reallocated in order to teach the latest medical disciplines and skills to keep pace with the propagating knowledge base of medical education [15,16]. Consequently, the use of cadavers in anatomy is declining and some institutions have gone so far as to eliminate dissection entirely [17,18], a decision made partially by the integration and advancement of computer assisted learning (CAL) tools. 

Challenges to anatomy education have led more than 20 veterinary schools to explore the use of animal alternatives in teaching [12]. Today’s students likely grew up in parallel with computers making them more dependent, highly accomplished, and appreciative of the value of CAL resources for learning [19,20]. Students describe CAL anatomy instruction as enjoyable [21,22,23] and accept computer-based interactive technology as an effective approach in conveying anatomical information [24,25]. Advantages of supplementing traditional methods with CAL include novel anatomic perspective, increased portability and longevity, and increased standardization [26,27]. 

Computer assisted learning tools are rapidly proliferating in production and curricular integration [6,28,29], yet most of the educational developments in virtual anatomy imaging have not been tested adequately for effect on student learning outcomes [10], and further research is necessary before it can be widely accepted [25,30,31]. Continued research will also assist instructors in determining which educational tools meet their teaching expectations and guide the production of valid novel CAL programs [32,33]. 

This study evaluated the outcomes and perceptions of students after supplementing their traditional methods of anatomy education (text references, lecture, and laboratory cadaver dissection) with a web based virtual anatomy program called IVALA™ (www.ivalalearn.com) [34]. It was hypothesized that students who supplemented the standard curriculum with the CAL program would have significantly improved examination scores and that the software would be well accepted by students. 

## 2. Materials and Methods 

### 2.1. IVALA™ Program 

The IVALA™ program was chosen for this study because of its free access to students through the online website *Veterinary Information Network*™ (www.vin.com) [35]. The IVALA™ program is an interactive virtual three-dimensional anatomy software provided as an online educational resource. Content in IVALA™ is created primarily from computerized tomography and magnetic resonance imaging data reconstructions that have been modified and enhanced. Students attain access to the program directly from a personal computer or tablet within a user’s web browser via an internet connection. Within the program, a virtual canine specimen may be rotated in all directions, zoomed, and panned. Individual structures within the virtual specimen may be selected to show identification as well as text description of the specific anatomic tissue. Important characteristics of each structure (such as origin, insertion, action, and its relevance to form and function) are emphasized. Each structure may be virtually removed to view underlying or associated structures. 

### 2.2. RUSVM Gross Anatomy 1 Course 

The study was conducted during the fall semester of 2016 at the Ross University School of Veterinary Medicine (RUSVM). All students were exposed to the same RUSVM 4 credit veterinary gross anatomy curriculum over a 15-week semester. The course consisted of 21 lecture hours and 64 hours of cadaver dissection laboratory. In addition to the two text books, *Guide to the Dissection of the Dog* [36] and *Textbook of Veterinary Anatomy* [37], all students were provided access to lecture power point presentations in both slide and audio/video formats. All students had access to relevant course notes and materials, including illustrated or photographic dissection guides containing labelled anatomic images and demonstration videos. No difference in anatomy course instruction was provided to participants of this study. No course content was covered in IVALA™ that was not covered in the course at the time of this study. Any conceptual knowledge was presented equally to all students through lectures and laboratory demonstrations. Of the four instructors associated with course instruction, only one had knowledge of who was in the experimental group. The course coordinator, who is not an author in this study, created the exams and oversaw all grading.

### 2.3. Participants 

Use of the IVALA program could not be restricted or regulated due to its online open access, so the experimental design was done on a voluntary and self-reported basis. Of the 179 students enrolled in the fall 2016 RUSVM first semester class, 93 (52%) students initially volunteered to be part of the intervention group; however only 56 (31.3%) ultimately reported having used IVALA™ for studying. Students who did not use IVALA™ to supplement their learning of anatomy were used as the control group (n = 123, 68.7%). IVALA™ users were asked to locate and appreciate a list of specific anatomic structures using IVALA™. The list of structures was taken directly from required learning objectives from the course curriculum for all students. These students were instructed to visualize each structure completely and recognize important characteristics as emphasized in curriculum (size, shape, origin, insertion, action, and its relevance to form and function). Tracking of student interaction with IVALA™ was completed via good faith student activity logbook. The frequency and time dedicated by each student toward studying with IVALA™ was self-governed.

All participants were asked to read and sign an informed consent before the start of the study. Guidelines and standards for the use of students as research subjects relative to this research were established and approved by RUSVM Institutional Review Board (IRB# 16-06-XP).

### 2.4. Survey Tools

Qualitative, and quantitative perception data was gathered by questionnaires administered before and after interactions with IVALA™, via a secure RUSVM website using the program E*Value™ [38]. Completion of the survey was voluntary. 

Survey questions were developed following the same principles as in assessing another CAL education tool by Linton et al., 2005 [39] and were created to address six central themes of investigation:

(1) What is the participant’s general attitude toward CAL and the IVALA™ program?
(2) What is the perceived educational value of CAL and the IVALA™ program in relation to other learning resources?
(3) What effect did IVALA™ have on the laboratory dissection experience?
(4) Was the accuracy, fidelity, and content of IVALA™ suitable for this anatomy course?
(5) Was the IVALA™ program user friendly?
(6) Was the IVALA™ program beneficial to anatomic education?

Students’ responses were captured using a 5-point Likert scale (1 = strongly disagree, 5 = strongly agree). Furthermore, an open-ended inquiry was included to collect comments surrounding the students’ feelings, experiences, questions or concerns regarding the use of the IVALA™ program.

### 2.5. Examinations 

Anatomy examination scores were used to evaluate and compare intervention and control group outcomes. Student outcomes of two examinations were the dependent variables, and the method of study was the independent variable. The first examination was a traditional laboratory practical comprised of 25 multiple choice and 20 fill in the blank style questions. The second examination was a written 30 multiple-choice question test. Only the practical examination utilized dissected specimens, which were tagged for reference to a specific question. Both examinations included questions that emphasized anatomic identification of structures, origins, insertions, and actions of muscles, as well as relationships between individual structures. The topic matter of these examinations included the bones, muscles, and joints of the thoracic and pelvic limbs, as well as axial skeleton of the canine and equine. The course instruction and content were inclusive of all examined material; however, the IVALA™ program content was not completely inclusive of the examined material; therefore, questions relevant to the program content could be evaluated separately from and in comparison to those questions that were not associated with IVALA™ content. On the first examination, 20 out of a total of 45 questions were associated with anatomy represented by the IVALA™ program, and the second examination included 11 out of a total of 30 questions associated with anatomy represented by the IVALA™ program. Questions which were not associated with used as the negative control across the cohort subgroups. Questions were of similar nature, content, and difficulty to previous semesters, and were developed by an anatomy instructor who was not associated with this study. 

### 2.6. Statistical Analysis 

All quantitative responses were cumulated using Microsoft Excel™, and evaluated using the software R™ [40]. The Shapiro test was used to determine dependent variable distribution, and was found to be not normal (*p* < 0.05). To compare examination scores between student groups a Wilcoxon signed-rank test was performed on outcomes from both examinations. The Wilcoxon test was used to compare examination scores between genders. A value of *p* < 0.05 was considered to be significant. 

Student open responses were analyzed for common themes using axial and open coding that is based on Grounded Theory [41,42,43,44,45]. This retrospective process involved the extraction of one or more points of interest from each written comment, with subsequent independent placement into a topic theme by two researchers. Repeated assessment of comments’ points of interest and their associated themes resulted in subdivision or pooling of themes into categories until each comment was appropriately allocated without redundancy. Two of the authors of this study independently participated and agreed on the coding to ensure reliability (W.B.L. and C.S.S.). 

## 3. Results

### 3.1. Examination Outcomes

Analysis of the mean of examination 1 scores from questions associated with IVALA™ content shows a significantly higher score amongst IVALA™ users in comparison to non IVALA™ users (*p* = 0.007). There was no significant difference between mean scores when evaluating questions not relevant to IVALA™ content. Overall, IVALA™ users scored higher than non IVALA™ users (*p* = 0.02).

The second examination had similar outcomes, with IVALA™ users performing better than non IVALA™ users overall (*p* = 0.01). When the IVALA™ associated questions were isolated, IVALA™ users scored higher than non IVALA™ users (*p* = 0.01). Similar to the first examination outcomes, when questions not associated with IVALA™ material were evaluated, no significant difference in mean score was noted. 

Only 8 students provided a record of their time spent studying with IVALA™. Unfortunately, student self-reporting of IVALA™ interaction times were inadequate, and as such, no conclusions could be drawn regarding a time dependent response to its use.

No significant difference in distribution of mean scores was noted between genders. A summary of all mean examination scores and the calculated Wilcoxon test p-values of exam score comparisons between IVALA™ users and non IVALA™ users are summarized in Table 1. 

### 3.2. Pre Interaction Survey

Of the 93 students who volunteered to participate in this study, 90 (98%) responded to the pre-interaction survey. Category means indicated that participants enjoyed computer-based learning (mean = 3.73), were interested in using IVALA™ (mean = 4.68). Participants agreed that there is no substitute for hands on experience, no matter how realistic a computerized simulation may be for learning (mean = 4.12). Overall, there was a strong agreement with the statement “I believe a mixture of traditional cadaver-based anatomy lessons parallel and complementary to computer-based learning would be the best possible experience for my education” (mean = 4.53). Cumulated data from the pre-interaction survey Likert scale responses are summarized in Table S1.

Thirty-four students responded to the open-ended inquiry, and a summary of coded comments are provided in Table S2. 

### 3.3. Post Interaction Survey

Of the 93 students who volunteered to participate in this study, 56 (60.3%) used IVALA and responded to the post exposure survey. Cumulated data from the post interaction survey Likert scale responses are summarized in Table S3. The general perception was that IVALA™ was enjoyable (mean = 3.81), recommended for future RUSVM anatomy courses (mean = 4.02), and beneficial to the knowledge of veterinary anatomy (mean = 3.70). Students responded that computerized learning tools like the IVALA™ program will be an essential part of anatomy education in the future (mean = 4.02); however, students disagreed with computerized learning completely replacing the use of canine cadavers for anatomic education (mean = 2.07). 

Students noted that the IVALA™ program exhibited accurate anatomy (mean = 4.09), looked realistic (mean = 3.74), and was suitable for learning the required anatomic structures for the RUSVM Gross Anatomy semester 1 course (mean = 3.67). There was a positive trend toward the IVALA™ program’s ease of use (mean = 3.60) and belief that manipulation of the IVALA™ virtual specimen allowed for an adequate view of each anatomic structure of interest (mean = 3.67). The coded results of students’ open-ended comments are provided in Table S4. 

## 4. Discussion

These results indicate that studying with the IVALA™ program improved student test scores. Specifically, higher mean scores were observed on both examinations of the IVALA™ users (mean = 77.1%) over non IVALA™ users (mean = 71.2%) from questions associated with IVALA™ material (*p* = 0.003). This, along with a lack of significant difference between student performances from control questions which were not associated with IVALA™ material supports previous literature linking supplemental computer aided learning with improved learning of gross anatomy [46]. 

Results indicate that deliberate practice with the IVALA™ program accompanied by well-defined learning objectives and focused, repetitive practice leads to improved outcomes. These findings substantiate previously documented benefits associated with simulation-based healthcare education over traditional teaching methodology alone [47].

Students in this study described their experience with the IVALA™ program as enjoyable, stated that the IVALA™ program was beneficial to their knowledge of veterinary anatomy, and would recommend the use of IVALA™ for future RUSVM anatomy students. This corroborates previous literature which found that CAL was well accepted in anatomical education [24,25,28,48] and is generally perceived as enjoyable by students [21,22,49].

This cohort of students perceived anatomy education utilizing cadavers and computers as complementary and their opinions were overwhelmingly opposed to the replacement of cadaveric dissection with CAL. These findings are consistent with research associated with other CAL resources [8,30]. Currently there is insufficient evidence that computer aided learning can replace current teaching methods entirely [1]. Like other research in the field of virtual anatomy simulation [25], our objective in this experiment was not to encourage replacement of cadaveric dissection, but rather to enhance traditional teaching methods. 

Considering only 56 of the initial 93 volunteers (60.3%) studied using the IVALA™, one can make the supposition that students will study to best fit their personal style and needs, regardless of directed study technique. This lack of uniformity of CAL use between anatomy students, as well as the student specific characteristics that are associated with this variability is supported elsewhere [50]. Self-selection bias that exists in this cohort leaves opportunity for further study. It would be especially interesting to know if the improvement in outcomes associated with IVALA use would be diminished if the student groups were created randomly and if students who did not chose to utilize IVALA would benefit from its use to the same extent of a self-selected cohort.

There are several limitations noted within this study. Specifically, comparison of study times between students who used IVALA™ and the control group was not possible because the control group did not volunteer to be regulated by this study. Time spent studying in coordination with IVALA™ compared to time spent utilizing traditional study methods, or the association between study time and group could also not be obtained. There is a possible association to a student’s likelihood of volunteering and his/her enthusiasm to a subject and therefore an inherent probability of higher scores regardless of study style, though this limitation is moderated considering the lack of significance between the mean scores of IVALA™ users, and non-users on questions not associated with IVALA™ material. We also understand that comparisons between IVALA™ users and non-users on questions not related to IVALA™ content result in *p* = 0.3 on exam 1, and *p* = 0.2 with relatively low power of 29% and 30% respectively, which shows that we should take this absence of difference with caution.

Previous research comparing computer aided learning and traditional teaching methods has generated conflicting results. Some studies have agreed with our findings and concluded that the study of anatomy with incorporation of new methodologies based on computer sciences are a benefit to the student [21,51,52,53]. Contrarily, other research indicates that computer-based learning resources may have significant disadvantages in learning nominal anatomy compared to traditional specimens [5,54]. These discordant findings provide evidence for a need to continue research in this subject. Appropriate utilization of anatomy teaching resources will be assisted by a further understanding of image perception by the brain as well as characteristics that influence a student’s three-dimensional learning [55].

## 5. Conclusions

This study provides evidence that IVALA™ is associated with improved student understanding and examination outcomes in veterinary gross anatomy. Perceptions of the IVALA™ software are generally positive, however, students do not support the replacement of cadaver dissection with CAL. This research demonstrates that virtual CAL software may enhance anatomy instruction resulting in improved student examination scores, and is positively perceived by students. Virtual anatomy tools provide an opportunity for veterinary medical education to support and enhance the learning of anatomy.

## Figures and Tables

**Table 1:** Mean examination scores (%) from IVALA and Non IVALA users and all examination question groups.

Student Group	Exam 1			Exam 2			Both Exam Scores Combined		
	IVALA™ associated questions	Non IVALA™ associated questions	All (total)	IVALA™ associated questions	Non IVALA™ associated questions	All (total)	IVALA™ associated questions	Non IVALA™ associated questions	All (total)
IVALA™ users (n = 56)	77.8	86.0	82.5	75.8	89.2	84.4	77.1	87.4	83.3
Non IVALA™ users (n = 123)	71.6	83.5	78.5	70.4	86.7	80.9	71.2	84.9	79.5
*p*-value comparisons	**0.007**	0.3	**0.02**	**0.01**	0.2	**0.01**	**0.003**	0.09	**0.006**

Significant *p*-values are bolded.

## Supplementary Materials

The following are available online at http://www.mdpi.com/2306-7381/5/2/58/s1, Table S1: Pre-interaction survey Likert response data (n = 91), Table S2: Coded responses to the Pre-Interaction open-ended question: Provide any comments about your feelings, expectations or concerns regarding your upcoming use of IVALA™. (n = 28), Table S3: Post interaction survey Likert response data. (n = 56); Table S4: Coded responses to the post interaction open-ended question: Provide any comments about your feelings, expectations or concerns regarding your use of IVALA™. (n = 24).
