# Supplementary material for: Computer Assisted Learning: Assessment of the Veterinary Virtual Anatomy Education Software IVALA™
Source: Vet Sci. 2018 Jun 19;5(2):58. doi: 10.3390/vetsci5020058 (PMC6024603; doi:10.3390/vetsci5020058)
Supplement: Supplementary file 1 [file vetsci-05-00058-s001.pdf]

Table S1. Pre-interaction survey Likert response data (n=91).

Category	Likert Scale Question	Median	Mean	Standard Deviation
Category 1	What is the general attitude and experience with CAL educational tools and the IVALA™ program?			
	I relied heavily on computer based resources during my previous education.	3	3.05	1.15
	I enjoy computer based learning.	4.00	3.73	0.65
	I am interested in using the IVALA™ program.	5.00	4.68	0.49
	I believe the IVALA™ "VITAL" program will be helpful to my learning, understanding, and retention of anatomic information.	5.00	4.54	0.52
Category 2	What is the perceived educational value of CAL tools in relation to other learning resources?			
	I would rather use computer based learning than paper text.	3.00	3.16	0.98
	I would rather use computer based learning than attend live lectures.	2.00	2.48	1.03
	I would rather use computer based learning than hands on experience of cadaver dissection.	2.00	1.88	0.88
	I would like to have more of my educational resources provided in computerized from, rather than traditional forms like text, live lectures, and laboratory exercises.	3.00	2.54	0.91
	I could happily do without computer based learning resources. I prefer traditional forms like text, live lectures, and laboratory exercises.	3.00	3.03	1.02
	I wish all of my learning resources were in hard copy text supplemented with hands on experience.	3.00	3.19	1.06
	I believe I would benefit from having all of my learning resources computerized.	2.00	2.62	1.00
	I think hands on experience through dissection of cadavers can be easily replaced by computerized resources.	2.00	2.02	0.80
	I think there is no substitute for hands on experience, no matter how realistic a computerized simulation may be for learning.	4.00	4.12	0.80
	I think computerized resources should be used as a supplement to traditional learning resources such as text, live lectures and laboratory exercises, but should never replace them.	4.00	4.30	0.77
	I would like the IVALA™ program (or a similar program) to completely replace traditional cadaver based learning in the future.	2.00	2.09	0.94
	I do not believe the IVALA™ program (or a similar program) could ever completely replace cadaver based anatomy education.	4.00	3.95	0.92
	I believe a mixture of traditional cadaver based anatomy lessons parallel and complementary to computer based learning would be the best possible experience for my education.	5.00	4.53	0.70

Likert Scale; 1 = strongly disagree, 2 = disagree, 3 = neutral, 4 = agree, 5 = strongly agree.

Table S2. Coded responses to the Pre-Interaction open-ended question: Provide any comments about your feelings, expectations or concerns regarding your upcoming use of IVALA™. (n = 28).

Comment category	Number of comments	Percentage of students providing a comment fitting this category
I believe there will be educational value in IVALA.	28	82.35
I am doubtful IVALA™ can replace cadaver dissection.	7	20.59
Hands on experience (dissection) is important to me.	4	11.76
I have concern for lack of fidelity or technical issues.	3	8.82
Computerized learning is important to me.	3	8.82
Traditional text is important to me.	2	5.88

Table S3. Post interaction survey Likert response data. (n = 56).

Category	Likert Scale Question	Median	Mean	Standard Deviation
Category 1	What is the general attitude and experience with CAL educational tools and the IVALA™ program?			
	I enjoyed using the IVALA™ program.	4	3.81	1.06
	I would recommend using the IVALA™ program in future RUSVM anatomy classes.	4	4.02	0.97
	If there were an application for my mobile device that was similar to the IVALA™ program, I would use it as a study aid.	5	4.28	1.06
Category 2	What is the perceived educational value of IVALA™ in relation to other learning resources?			
	I believe computerized learning tools like IVALA™ program and other virtual learning tools will be an essential part of anatomy education for future students.	4	4.05	0.77
	I believe computerized learning models and simulation could completely replace the use of canine cadavers for anatomic education in the future.	2	2.07	1.12
	There are already enough computerized anatomy learning resources available for study and I feel that the addition of the IVALA™ program was a burden to my study load.	2	2.02	0.92
	I would have preferred to spend more time with the IVALA™ program rather than some of the other computer based resources provided for the veterinary anatomy course.	3	3.30	0.84
	I felt that I did not have enough time to spend with the IVALA™ program because there are so many other anatomy learning resources provided in the veterinary anatomy course.	3	2.82	1.07
Category 3	What effect did IVALA™ have on laboratory dissection experience?			
	Use of the IVALA™ software made me more confident during laboratory dissection of the cadaver dog.	3	3.27	1.10
	Use of the IVALA™ software made me more efficient during laboratory dissection of the cadaver dog	3	3.30	1.07
	The IVALA™ program made my dissection of the cadaver dog more clear and understandable.	4	3.56	1.05
Category 4	Was the accuracy, fidelity and content of IVALA™ suitable for this anatomy course?			
	The IVALA™ Virtual specimen looked realistic enough to learn the required anatomy effectively.	4	3.74	0.95
	The IVALA™ Virtual specimen exhibited accurate anatomy.	4	4.09	0.79
	The IVALA™ virtual specimen is suitable for learning the required anatomic structures for Gross Anatomy semester 1 course (VMA5113).	4	3.67	1.02
	I would like to see the IVALA™ program brought to a higher level of fidelity and accuracy.	4	3.84	0.84
	The IVALA™ program obviously lacks the detail of a cadaver canine specimen and I found these imperfections to be a significant hindrance to my learning.	2	2.42	0.86
	The current version of IVALA™ software has the accuracy and fidelity I needed to understand and learn the relevant canine anatomy of the forelimb and hind limb.	4	3.63	0.94
Category 5	Was the IVALA™ program user friendly?			
	The IVALA™ software was easy to use.	4	3.60	1.16
	Manipulation of the IVALA™ virtual specimen allowed for adequate view of each anatomic structure of interest.	4	3.67	1.02
	The IVALA™ virtual specimen was helpful because I could use it whenever / wherever I wanted (as long as I had internet service and access to a computer).	4	4.14	1.01
Category 6	Was the IVALA™ program beneficial to anatomic knowledge?			
	The IVALA™ program was beneficial to my knowledge of veterinary anatomy.	4	3.70	1.15
	Even though the fidelity and accuracy of the IVALA™ program was not perfect, I found its use to be beneficial to my overall knowledge.	4	3.93	0.82

Likert Scale; 1 = strongly disagree, 2 = disagree, 3 = neutral, 4 = agree, 5 = strongly agree.

Table S4. Coded responses to the post interaction open-ended question: Provide any comments about your feelings, expectations or concerns regarding your use of IVALA™. (n = 24).

Comment category	Number of comments	Percentage of students providing a comment fitting this category
IVALA™ was benefit to learning of anatomy	13	54.2
I had technical issues with IVALA™	8	33.3
The accuracy and/or fidelity of IVALA™ needs to be improved	4	16.7
IVALA™ is not a valid substitute for dissection	4	16.7
I would like this program for the iPad	3	12.5
I found other computerized programs to be more helpful	2	8.3
I would like IVALA™ to have quiz capability	1	4.2
I needed more guidance	1	4.2
I appreciate interaction of IVALA	1	4.2
